# The efficacy of contrast protocol in hepatic dynamic computed tomography: multicenter prospective study in community hospitals

**DOI:** 10.1186/2193-1801-2-367

**Published:** 2013-07-31

**Authors:** Masahiro Okada, Hiroshi Kondo, Hironobu Sou, Takamichi Murakami, Masayuki Kanematsu, Tomoaki Ichikawa, Shushi Yoshikawa, Kazuhito Shiosakai, Akiko Hayakawa, Kazuo Awai, Kengo Yoshimitsu, Yasuyuki Yamashita

**Affiliations:** Department of Radiology, Kinki University Faculty of Medicine, 377-2, Ohno-Higashi, Osaka-Sayamashi, Osaka, 589-8511 Japan; Department of Radiology, Gifu University Hospital, 1-1 Yanagido, Gifu, Gifu, 501-1194 Japan; Department of Radiology, University of Yamanashi, 1110 Shimokato, Chuo, Yamanashi, 409-3898 Japan; Central Radiology Department, Osaka Medical College, 2-7 Daigaku-chou, Takatsuki, Osaka, 556-8686 Japan; Clinical Data and Biostatistics Department, R&D Division, DAIICHI SANKYO CO., LTD, 1-2-58, Hiromachi, Shinagawa-ku, Tokyo, 140-8710 Japan; Medical Affairs Department, Business Intelligence Division, DAIICHI SANKYO CO., LTD, 3-5-1, Nihonbashi honcho, Chuo-ku, Tokyo, 103-8426 Japan; Department of Diagnostic Radiology, Institute of Biomedical Sciences, Hiroshima University, 1-2-3 Kasumi, Minami-ku, Hiroshima, 734-8551 Japan; Department of Radiology, Faculty of Medicine, Fukuoka University, 7-45-1, Nanakuma, Jonan-ku, Fukuoka, 814-0180 Japan; Department of Diagnostic Radiology, Graduate School of Medical Sciences, Kumamoto University, 1-1-1, Honjo, Kumamoto, 860-0811 Japan

**Keywords:** CT, Liver, Contrast media, Hepatocellular carcinoma, Injection method

## Abstract

**Purpose:**

To investigate four different contrast protocols to detect hypervascular hepatocellular carcinoma (HCC) most adaptable for patients at any body weight (BW) in clinical practice.

**Materials and methods:**

A post-marketing surveillance of liver dynamic CT was prospectively performed by four different protocols in 415 patients: Protocol-A, BW-tailored dose of contrast media (CM: iohexol 300 mgI/mL), fixed injection duration (30s), fixed scan timing at arterial phase (AP); Protocol-B, BW-tailored dose of CM, fixed injection duration (30s), by bolus tracking; Protocol-C, BW-tailored dose of CM, fixed injection flow rate, by bolus tracking; Protocol-D, 100 mL constant of CM at any BW, fixed scan timing. Scan timing and tumor conspicuity at AP was scored qualitatively. The quantitative CT values of aorta and tumor liver contrast (TLC) were obtained.

**Results:**

The qualitative rate assessed “good” as scan timing of AP in Protocol-C was significantly lower than those in Protocols A and D (difference:16.6%, 17.4%, *P* = 0.0069, *P* = 0.0140, respectively). Scatter plot of Protocol-D (R^2^ = 0.1283) at AP showed significant inverse relationship between TLC and BW (*P* =0.0053), although not significant in Protocols A, B, C.

**Conclusion:**

In patients with higher BW, protocols of BW-tailored dose of CM and/or fixed injection duration have no dependence on BW to diagnose hypervascular HCCs.

**Electronic supplementary material:**

The online version of this article (doi:10.1186/2193-1801-2-367) contains supplementary material, which is available to authorized users.

## Introduction

Multidetector-row computed tomography (MDCT) has allowed imaging of the liver in detail, because of the improved spatial and temporal resolution. But the optimal techniques for intravenous (IV) injection of contrast media (CM) and scanning of the liver to diagnose hepatocellular carcinoma (HCC) have been a subject of controversy for several years.

HCC is one of the most common malignancies worldwide. The majority of HCCs develop in cirrhotic livers, thus the early detection and characterization of this entity is important for decisions on therapeutic strategy. But, rapid scan speed with a MDCT scanner increases the difficulty to image hypervascular HCC during the arterial phase (AP) after CM injection. Therefore, we should know the optimal scanning protocol to start in the AP after IV injection of CM.

There are several important technical factors for the injection of CM, such as the dose (i.e. volume and concentration), injection rate, injection duration, body weight (BW) and scan delay time in the AP. The volume of CM, the concentration and the injection rate are directly related to maximum liver enhancement (Dean et al. 1980; Berland & Lee 1988; Claussen et al. 1984; Yagyu et al. 2005), whereas patients’ BWs are inversely related (Kormano et al. 1983). Some investigators have suggested that a minimum enhancement of 50 Hounsfield Units (HU) is necessary as adequate liver enhancement to obtain high conspicuity of low-attenuated hepatic lesions (Walkey 1991; Brink et al. 1995; Heiken et al. 1995). The total dose of CM is one of the most important technical factors to determine the amplitude of the contrast enhancement of the liver, because a fixed dose of CM provides different effects in patients with various BWs. Whereas, when we use the same fractional/total dose of iodine for the depiction of hypervascular HCC, rapid injection of CM with moderate concentration is more effective than is high concentration of CM (Awai et al. 2004a; Han et al. 2000). These facts are also shown using the theoretical compartmental model of (Bae et al. 1998a; Bae et al. 1998b) and the observations of (Awai et al. 2004b; Awai & Hori 2003). The concentration and injection rate of CM are important for determining the amplitude of contrast enhancement in hypervascular HCCs during AP. Moreover, the injection duration is also important to predict peak enhancement time in the liver, because it may be the only factor to restrict temporal changes in contrast enhancement. When a tailored dose of CM according to patients’ BW is used, a fixed injection duration method allows the minimization of the variation in aortic peak enhancement time for each patient (Ichikawa et al. 2006; Erturk et al. 2008). To achieve adequate liver enhancement for all patients with a wide variety of BW on CT, recent clinical studies have suggested that the dose of CM should be tailored according to patients’ BWs to obtain adequate liver enhancement (Awai et al. 2004b; Awai & Hori 2003; Kondo et al. 2008). The variation in liver enhancement among patients with different BWs is cancelled by using the BW-tailored dose of CM (Awai et al. 2004b). Thus, a tailored dose of CM according to patients’ BW has been a recent trend in hepatic dynamic CT protocols.

On the other hand, to achieve optimal detection of hypervascular HCC during the AP in liver dynamic CT, it is important to predict the peak time of aortic enhancement, because blood is supplied to tumors from the hepatic artery (which is a branch of the abdominal aorta) in patients with hypervascular HCC. The routine use of computer-assisted bolus tracking techniques (i.e. SmartPrep®) for AP scanning is recommended to detect hypervascular HCC (Tomemori et al. 2001). The imaging by bolus tracking technique is useful to catch the optimal scan timing during AP in patients with severe cardiac dysfunction.

Although these fingings above, the fixed injection rate of CM has been used in dynamic CT protocols of the liver rather than the fixed injection duration. Moreover, some radiologists use a uniform dose of intravenous CM in patients undergoing hepatic dynamic CT, although experimental data have indicated an inverse relationship between hepatic contrast enhancement on CT and patient BW (Kormano et al. 1983). These may be still the actual situations in some hospitals even nowadays in Japan. Moreover, the condition of the bolus tracking system settings for rapid scan speed with a MDCT scanner had not been standardized and optimized in community hospitals in Japan, although bolus tracking techniques were introduced into the daily clinical settings in community hospitals.

To our knowledge, no prospective study has systematically evaluated the role of CM injection protocols, including the dose (i.e. volume and concentration), injection rate, injection duration of CM, BW and scan delay time in the AP for hepatic dynamic CT for daily clinical settings in community hospitals.

Therefore, the aim of this study was to investigate most adaptable protocol for patients at any BW among the four injection protocols routinely used in community hospitals in Japan, by focusing the imaging of AP, which is thought to be the most important for liver dynamic CT to detect HCC.

## Materials and methods

### Patients

Since this study was non-interventional study conducted as post-marketing surveillance in Japan, in which the data is collected from daily clinical settings in each hospitals, the hospitals using either one of the following 4 injection protocols routinely used in Japan shown in the “Imaging methods” section were selected and signed contract with each hospital for participation in the study. During the period between June 2010 and November 2010, the study of liver dynamic CT was conducted by Daiichi Sankyo Co., Ltd., in accordance with the Good Post-marketing Study Practice (MHLW Ordinance No. 171 issued on December 20, 2004) in the 91 community hospitals in Japan. After receipt of signed contract for participation in the study from the 91 hospitals, consecutive patients who fulfilled the following inclusion criteria but no exclusion criteria were enrolled under the conditions of the daily clinical setting, until the target number of patients was reached. Liver dynamic CT was performed for 419 patients registered in each hospital, and all patients received one of 4 injection protocols shown in the “Imaging methods” section. The inclusion criteria were: (a) type B or C hepatitis or liver cirrhosis; (b) known HCC (up to 3 cm in size) without any treatment after the diagnosis from tumor biopsy, CT during arterial portography (CTAP) and CT during hepatic arteriography (CTHA), lipiodol CT, contrast enhanced magnetic resonance imaging with such as gadodiamide (Omniscan®, Daiichi Sankyo Co., Ltd. Tokyo, Japan), or gadoxetic acid (Primovist®; Bayer HealthCare Pharmaceuticals, Berlin, Germany), contrast enhanced sonography; (c) alternatively, patients in whom enlargement of tumor or increased markers for liver cancer alpha-fetoprotein and protein induced by vitamin-K absence II was observed; (d) patients who gave informed written consent; and (e) patients who underwent liver dynamic CT with 16 detectors-rows or 64 detectors-rows of MDCT for the assessment of HCC.

The exclusion criteria were: (a) patients with hypersensitivity to iodine CM; (b) patients with severe thyroid diseases; (c) patients with severe renal dysfunction; (d) patients with metastatic liver diseases; (e) patients with therapeutic conditions, such as radiofrequency ablation (RFA) or transarterial chemo-embolization (TACE) - within 3 months before CT examination; and (f) patients with severe fatty liver diseases, who had higher density of intrahepatic vessels compared to liver parenchyma on unenhanced CT.

**Figure 1:**
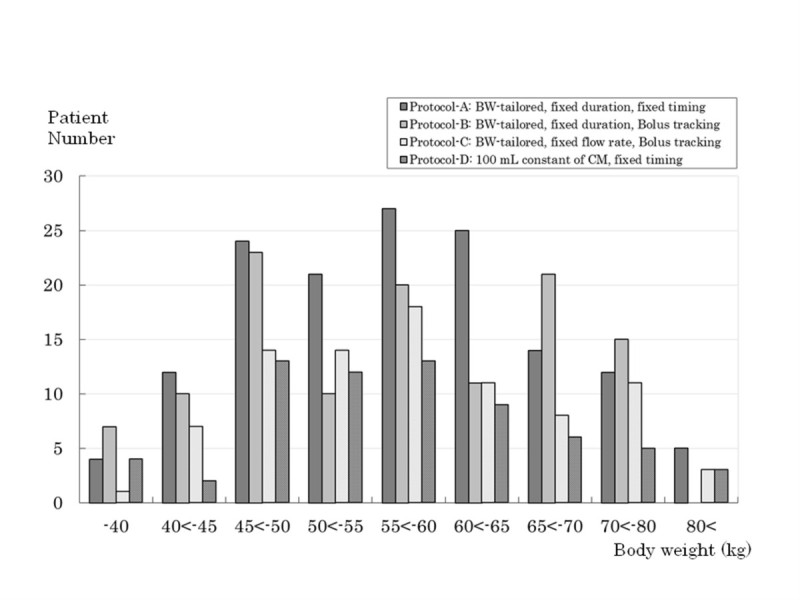
**Subjects’ body weight (BW) distribution.** There was no significant difference in weight distribution between each group of four protocols. -40; up to 40 kg of BW, 40 < −45; greater than 40 kg, up to 45 kg of BW, 45 < −50, 50 < −55, 55 < −60, 60 < −65, 65 < −70 and 70 < −80 are shown in similar to 40 < −45, 80<; greater than 80 kg of BW.

Four of 419 patients were excluded because of the following reason: two patients were with severe fatty liver judged by three reviewers of qualitative analysis as the exclusion criteria of this study, one patient was not administered iohexol but other iodine component that differ from the structured study protocol, and one patient was found to have been enrolled outside the contract period. Thus final study population consisted of 415 patients. Subjects’ characteristics are shown in Table 1. The background, characteristics and diagnosis methods of HCC are shown in Table 2. Of 415 patients, 340 had hypervascular HCC and remaining 75 had hypovascular HCC. These were from 1–3 HCCs in 318 patients, 4–6 HCCs in 35 patients, 7–9 HCCs in 14 patients and more than 10 HCCs in 48 patients. No obvious imbalance in gender, age and body weight was observed between the four protocols. There was no significant difference in weight distribution between each group of the four protocols (Figure 1).

**Table 1:** **Subjects’ characteristics**

		Protocol-A	Protocol-B	Protocol-C	Protocol-D	Overall
		(n = 144)	(n = 117)	(n = 87)	(n = 67)	(n = 415)
**Gender**	Male	111 (77.1)	79 (67.5)	65 (74.7)	45 (67.2)	300 (72.3)
	Female	33 (22.9)	38 (32.5)	22 (25.3)	22 (32.8)	115 (27.7)
**Age**		73 ± 8.9	73 ± 8.9	72 ± 8.6	71 ± 9.8	72 ± 9.0
**BW**		58.6 ± 12.0	57.4 ± 10.8	59.0 ± 10.7	57.4 ± 11.6	58.1 ± 11.3

Note; Data presented as number (%) of patients or mean ± standard deviation (SD); BW, body weight.

**Table 2:** **Background, characteristics and diagnosis methods for hepatocellular carcinoma**

		Protocol-A	Protocol-B	Protocol-C	Protocol-D
Background*					
Hepatitis B		5 (3.5)	8 (6.8)	6 (6.9)	6 (9.0)
Hepatitis C		36 (25.0)	35 (29.9)	17 (19.5)	19 (28.4)
LC		104 (72.2)	75 (64.1)	65 (74.7)	42 (62.7)
LC-B		9 (6.3)	8 (6.8)	3 (3.4)	2 (3.0)
LC-C		75 (52.1)	51 (43.6)	45 (51.7)	28 (41.8)
LC-Alcoholic		13 (9.0)	9 (7.7)	11 (12.6)	13 (19.4)
NASH		5 (3.5)	1 (0.9)	0.(0.0)	0 (0.0)
Others		5 (3.5)	9 (7.7)	8 (9.2)	2 (3.0)
Fatty liver		1 (0.7)	0 (0.0)	3 (3.4)	2 (3.0)
Others		2 (1.4)	2 (1.7)	0 (0.0)	0 (0.0)
**Characteristics**					
Size of tumor** (mm)		17.08 ± 6.42	16.85 ± 5.88	16.70 ± 6.27	16.60 ± 6.29
Range		3.0-30.0	5.6-30.0	7.0-30.0	6.6 − 29.0
	<20 mm	89 (61.8)	73 (62.4)	62 (71.3)	45 (67.2)
	>20 mm	55 (38.2)	44 (37.6)	25 (28.7)	22 (32.8)
Number of tumors	1-3	109 (75.7)	97 (82.9)	65 (74.7)	47 (70.1)
	4-6	14 (9.7)	8 (6.8)	7 (8.0)	6 (9.0)
	7-9	6 (4.2)	3 (2.6)	1 (1.1)	4 (6.0)
	>10	15 (10.4)	9 (7.7)	14 (16.1)	10 (14.9)
Diagnosis methods*					
Biopsy		5 (3.5)	1 (0.9)	0 (0.0)	0 (0.0)
CE-MRI		33 (22.9)	39 (33.3)	31 (35.6)	14 (20.9)
CTAP/CTHA		10 (6.9)	9 (7.7)	10 (11.5)	9 (13.4)
Angiography		29 (20.1)	43 (36.8)	10 (11.5)	26 (38.8)
Lipiodol CT		12 (8.3)	15 (12.8)	4 (4.6)	3 (4.5)
Sonography		42 (29.1)	56 (47.9)	40 (46.0)	28 (41.8)
Growth of tumor		45 (31.3)	27 (23.1)	37 (42.5)	11 (16.4)
Increased tumor markers		78 (54.2)	70 (59.8)	45 (51.7)	34 (50.7)

Note; Data presented as number (%) of patients or mean ± standard deviation (SD);

LC, Liver cirrhosis; NASH, non-alcoholic steatohepatitis, CE-MRI, Contrast-enhanced magnetic resonance imaging;

CTAP, computed tomography during arterial portography; CTHA, computed tomography during hepatic arteriography

** Background and diagnosis methods were made by multiple selections.

* Tumor selected as target tumor for quantitaive analysis.

### Imaging methods

Dynamic CT protocols in this study were selected by the daily work of each hospital. Four injection protocols were employed for the study as follows: Protocol-A, BW-tailored dose of CM (300 mgI/mL of iohexol, Omnipaque® 300, Daiichi Sankyo Co., Ltd.), fixed injection duration (30 s), fixed scan timing at AP; Protocol-B, BW-tailored dose of CM, fixed injection duration (30 s), scan timing at AP adjusted by bolus tracking; Protocol-C, BW-tailored dose of CM, fixed injection flow rate, scan timing at AP adjusted by bolus tracking; Protocol-D, 100 mL constant of CM at any BW, fixed scan timing at AP. Iodine dose per weight for BW-tailored dose of CM in Protocols A, B and C, fixed scan start time at AP in Protocols A and D, trigger and scan delay for AP by bolus tracking in Protocols B and C, and fixed injection flow rate in Protocol-C were decided as a clinical CT examination in each hospital. In terms of the average, the dose of CM per weight was higher in Protocols A and C, and injection flow rate was highest in Protocol-A. Injection duration in Protocols C and D with fixed flow rate was longer than 30 s, although the injection duration was fixed as constant at 30 s in Protocols A and B (Table 3). The delay time of AP from the initiation of CM injection was recorded. In Protocol-B (mean ± SD / median, 34.92 ± 4.85 / 35.0 s) and C (32.77 ± 5.48 / 33.0 s) using bolus tracking were earlier compared to those in Protocols A (38.87 ± 2.95 / 40.0 s) and D (38.27 ± 4.19 / 40.0 s).

**Table 3:** **Injection data of contrast material**

	Protocol-A	Protocol-B	Protocol-C	Protocol-D
**Total dose (mL)**	113.9 ± 20.42	109.3 ± 21.87	116.3 ± 19.89	100.0
**Dose per weight (mL/kg)**	1.97 ± 0.275	1.90 ± 0.132	1.98 ± 0.138	1.81 ± 0.369
**Iodine dose per weight (mgI/kg)**	589.6 ± 82.35	571.3 ± 39.61	593.9 ± 41.35	543.6 ± 110.7
**Injection flow rate (mL/sec)**	3.79 ± 0.681	3.64 ± 0.729	3.44 ± 0.334	3.21 ± 0.243
**Injection duration (sec)**	30.0	30.0	33.9 ± 5.51	31.3 ± 2.17

Note; Data presented as mean ± standard deviation (SD).

Our study data were obtained from 99 CT machines (Toshiba 44, General Electric 32, Siemens 13, Philips 10) in 91 hospitals. Fixed 5 mm slice thickness of CT images was employed in all hospitals. And other CT imaging parameters, such as detector-row, tube voltage and tube current, are shown in Table 4. Liver dynamic CT images were collected as electronic data (DICOM standard) with clinical information including patient background from each hospital.

**Table 4:** **Imaging methods for each computed tomography protocol**

		Protocol-A	Protocol-B	Protocol-C	Protocol-D
**Detector-rows**	16	13 (33.3)	4 (12.9)	6 (40.0)	4 (28.6)
	64	26 (66.7)	27 (87.1)	9 (60.0)	10 (71.4)
**Tube voltage**	<120KV	1 (2.6)	0 (0.0)	0 (0.0)	0 (0.0)
	120KV	36 (92.3)	29 (93.5)	15 (100.0)	14 (100.0)
	>120KV	2 (5.1)	2 (6.5)	0 (0.0)	0 (0.0)
**Tube current**	Auto	33 (84.6)	29 (93.5)	14 (93.3)	14 (100.0)
	Fixed	6 (15.4)	2 (6.5)	1 (6.7)	0 (0.0)
**Scan speed**	<0.5	4 (10.3)	2 (6.5)	1 (6.7)	0 (0.0)
	0.5	20 (51.3)	26 (83.9)	12 (80.0)	11 (78.6)
	0.5<	14 (35.9)	2 (6.5)	2 (13.3)	3 (21.4)
	Unknown	1 (2.6)	1 (3.2)	0 (0.0)	0 (0.0)

Note; Data presented as number (%) of CT machines; KV, kilovolt.

### Imaging evaluation

#### Qualitative analysis

In order to ensure the reliability of reviewers, three independent reviewers, who had more than 13 years of experience in liver CT, had a training of the judgment for the liver dynamic CT to make criteria and consensus of qualitative evaluation. After that, three reviewers evaluated all imaging data in the points of scan timing of AP and the degree of tumor conspicuity of HCC in the AP.

On the actual evaluation, the information on the patient background, specific institutions and the imaging techniques were blinded for reviewers. When reviewing by three independent reviewers, the display setting of liver dynamic CT images, such as window width (WW) and window level (WL), was fixed as WW 270 and WL 60. The efficacy of imaging in the diagnosis of HCC in the AP of each liver segment was evaluated by the 3 independent reviewers, and classified into 4 grades as tumor conspicuity: “excellent (HCC enhancement at AP ≥ the enhancement of aorta)”, “good (liver parenchyma < < HCC enhancement at AP < the enhancement of aorta)”, “fair (liver parenchyma < HCC enhancement at AP < < the enhancement of aorta)” and “poor (liver parenchyma ≥ HCC enhancement at AP)”. These gradings were employed for the largest target 10 tumors. The scan timing of AP was investigated by these 2 grades: “good (arterial and portal vein enhancement of the liver are seen, but hepatic vein enhancement is not seen)”, “poor (portal vein enhancement is not seen, or both portal vein and hepatic vein in the liver are seen)”. The evaluation result was adopted when ratings given by 2 reviewers were consistent. When ratings by 3 reviewers were completely inconsistent, the case was discussed to determine the final rating.

#### Quantitative analysis

CT values (mean ± SD) in HU were measured and recorded by physicians at each institution site as follows; The mean attenuation of the abdominal aorta at the level of main portal vein [region of interest (ROI) size, approx. 1.0 cm^2^], hepatic parenchyma at the level of main portal vein (three ROIs in the left lobe, anterior section of right lobe and posterior section of right lobe of the liver; each ROI size, approx. 2.0 cm^2^), and HCC (ROI size, approx. 0.5 cm^2^) by using the CT attenuation values. ROIs of liver parenchyma were placed not to cover liver tumor region, visible blood vessel structure, bile ducts, calcified areas and artifacts. The target HCC was selected among the tumors within 3 cm in cases where there were multiple regions. And HCC enhancement was evaluated in the most enhanced portion of the tumor, when heterogeneous enhancement of HCC existed. The enhancement effect at AP was calculated as the absolute difference in the attenuation values of the abdominal aorta at unenhanced scanning versus AP of contrast-enhanced scanning. When automatic tube current modulation was used, data of SD values exceeding twice the setting SD value were excluded. Finally, the following parameters were calculated and used for the analysis:

Aortic enhancement (HU) = density of aorta at AP (HU) – density of aorta at unenhanced phase (HU)

The tumor conspicuity of HCC can be expressed by the attenuation differences between HCC and surrounding hepatic parenchyma - that is, tumor liver contrast (TLC).

$$\begin{array}{l} \mathrm{TLC}=\text{density}\;\mathrm{of}\;\text{target}\;\mathrm{tumor}\;\mathrm{at}\;\mathrm{AP}\;\left(\mathrm{HU}\right)\\ \;–\;\text{density}\;\mathrm{of}\;\text{hepatic}\;\text{parenchyma}\;\mathrm{at}\;\mathrm{AP}\;\left(\mathrm{HU}\right) \end{array}$$

Scatter plot of the relationship between BW and TLC at AP for each protocol was made to investigate the degree of linear approximation.

### Safety

Two of 419 patients were excluded because of the following reason: one patient was not administered iohexol but other iodine component that differ from the structured study protocol, and one patient was found to have been enrolled outside the contract period. Finally, the investigation into the safety of 300 mgI/mL of iohexol was performed in remaining 417 patients. The adverse reaction and the extravasation were evaluated by physicians at each institution site.

### Statistical analysis

For evaluation of the efficacy of imaging at AP, the best rating given to segments obtained from each patient was considered as the final rating for the patient. Moreover, patients rated as “excellent” or “good” were defined as those rated as “excellent or good”. The proportion of patients who were rated as “excellent or good” in each imaging protocol was compared by using a linear probability model. To compare aortic enhancement and quantitative TLC among the four imaging protocols, an analysis of variance model was performed. Moreover, to investigate the efficacy of 4 protocols in each BW range, tumor conspicuity and quantitative TLC at AP were compared according to the BW, which was divided into three subgroups (< 50 kg vs. 50–60 kg vs. > 60 kg), and the relationship between BW and TLC at AP was shown with scatter plots and the regression line by each protocol.

A P value less than 0.05 was considered to indicate statistical significance and all analyzes were performed by using SAS® System Release 8.2 (SAS Institute Inc., Carey, NC, USA). Since this study was exploratory study, sample size was determined without statistical consideration and no adjustment for multiplicity was performed.

## Results

### Qualitative analysis

**Figure 2:**
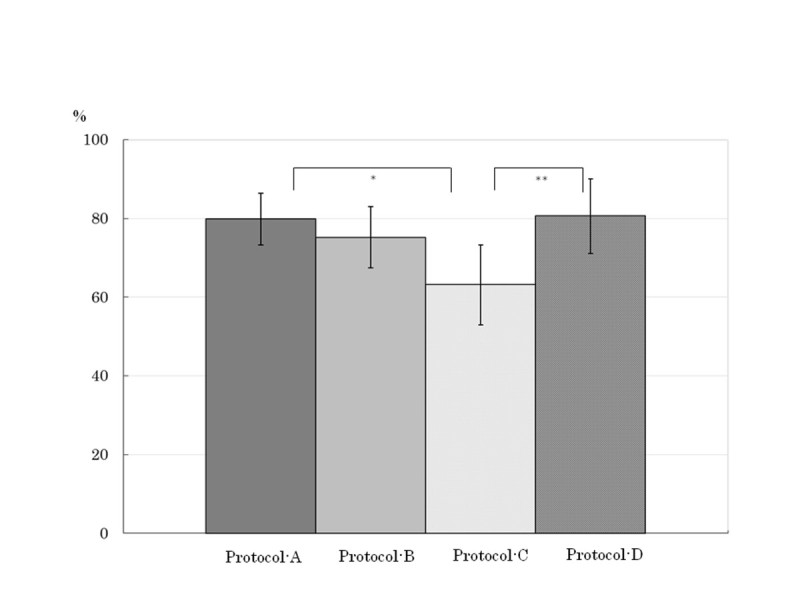
**Qualitative rate assessed “good” as scan timing at arterial phase (AP).** The qualitative rate assessed “good” as scan timing at AP in Protocol-C was significantly lower than those in Protocol-A (*; *P* = 0.0069) and D (**; *P* = 0.0140 ). Error bars indicate 95% confidence interval.

**Figure 3:**
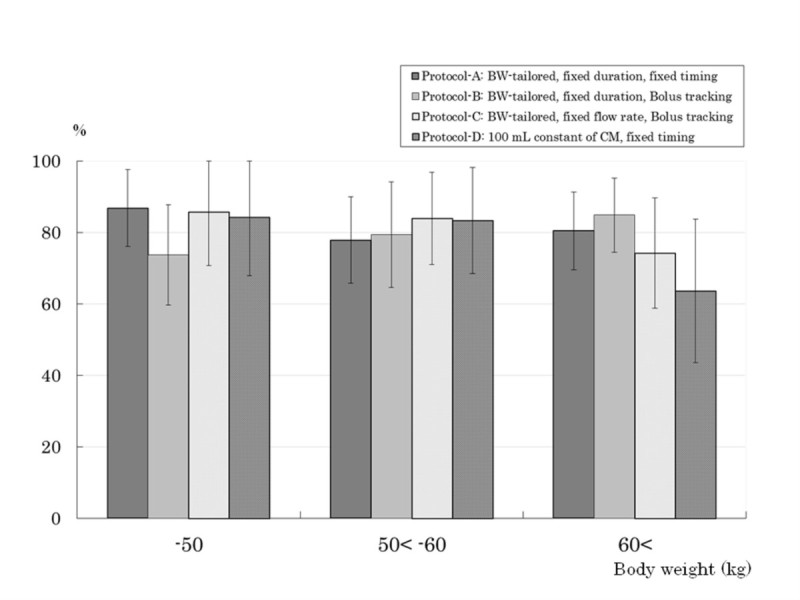
**The rate of patients rated as “excellent or good” in the qualitative tumor conspicuity at arterial phase (AP).** Regarding qualitative evaluation, tumor conspicuity of hepatocellular carcinoma obtained in the subgroup of body weight (BW) > 60 kg was not discrepant with the quantitative evaluation of the aorta enhancement (Figure 4). In the subgroup of BW > 60 kg, tumor conspicuity in Protocol-D tended to be lower than those in the other groups, and the highest tumor conspicuity was obtained in Protocol-B, which included fixed duration with bolus tracking. In the subgroups of BW ≤ 50 kg, tumor conspicuity in Protocol-B tended to be lower than those in the other groups. Error bars indicate 95% confidence interval. -50; up to 50 kg of BW, 50 < −60; greater than 50 kg, up to 60 kg of BW, 60<; greater than 60 kg of BW.

The qualitative rate assessed “good” as scan timing at AP in Protocol-C was significantly lower than those in Protocols A (difference: 16.6%, *P* = 0.0069) and D (difference: 17.4%, *P* = 0.0140) (Figure 2). The qualitative rates assessed “excellent or good” as tumor conspicuity at AP were Protocol-A, 81.3%, Protocol-B, 79.6%, Protocol-C, 80.7%, and Protocol-D, 76.9%. In patients with higher BW (> 60 kg), qualitative tumor conspicuity at AP in Protocols A and B were higher than those in Protocol-D (difference: 16.8%, *P* = 0.1509, 21.1%, *P* = 0.0669, respectively), and the highest tumor conspicuity was obtained in Protocol-B, which was fixed duration with bolus tracking (Figure 3). Whereas, in the subgroup of BW ≤ 50 kg, tumor conspicuity in Protocol-B showed the lowest value of the 4 protocols.

### Quantitative analysis

**Figure 4:**
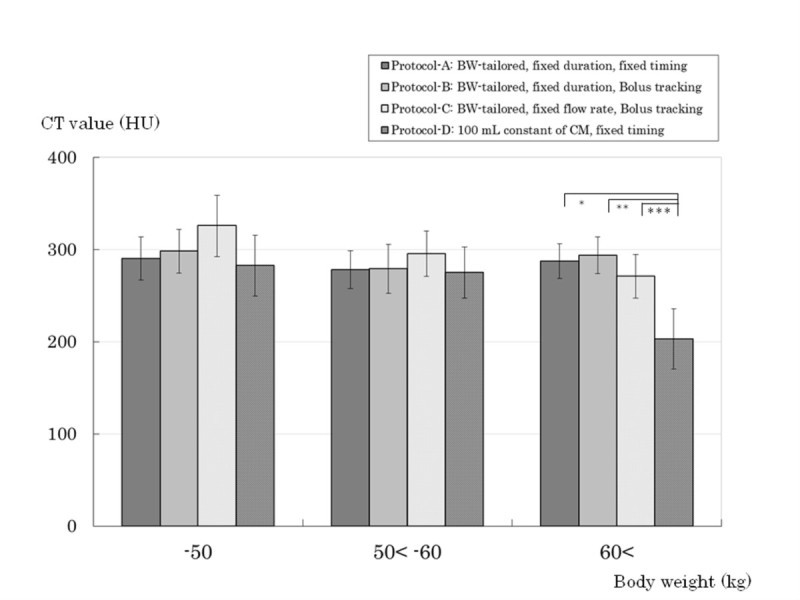
**Quantitative aortic enhancement at arterial phase (AP).** The timing of AP in Protocols B and C was early, so that contrast enhancement of the aorta was better than with the other protocols. It is suitable for the contrast imaging of the aorta in Protocols B and C. In the subgroup of body weight (BW) > 60 kg, the amount of iodine especially in Protocol-D was less than those in the other groups, and the aortic enhancement was significantly lower in Protocol-D by analysis of variance. (*; *P* < 0.001, **; *P* < 0.001, *** *P* = 0.0011). In the subgroups of BW ≤ 50 kg, the aortic enhancement in Protocol-C tended to be higher than those in the other groups. Error bars indicate 95% confidence interval. CT; computed tomography, HU; Hounsfield unit. -50; up to 50 kg of BW, 50 < −60; greater than 50 kg, up to 60 kg of BW, 60<; greater than 60 kg of BW.

**Figure 5:**
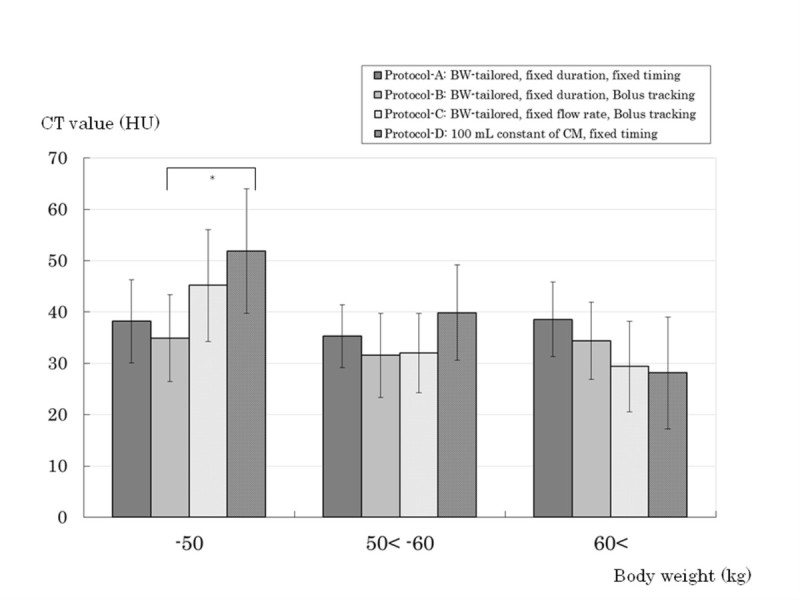
**Quantitative tumor liver contrast (TLC) at arterial phase (AP).** Regarding quantitative evaluation, the subgroup of body weight (BW) ≤ 50 kg in Protocol-D showed a significantly higher TLC of hepatocellular carcinoma compared with Protocol-B by analysis of variance. (*; *P* = 0.0249, because it is considered that an excessive amount of iodine was administered to patients with low BW in Protocol-D). In the subgroup of BW > 60 kg, the TLC in Protocol-D was less than those in the other groups. Error bars indicate 95% confidence interval. CT; computed tomography, HU; Hounsfield unit. -50; up to 50 kg of BW, 50 < −60; greater than 50 kg, up to 60 kg of BW, 60<; greater than 60 kg of BW.

In the subgroup of BW > 60 kg, the aortic enhancement was significantly lower in Protocol-D (the amount of iodine especially in Protocol-D was less than in the other groups). Subgroups of 50 kg < BW ≤ 60 kg and BW ≤ 50 kg did not show significant differences in the aortic enhancement between the 4 protocols (Figure 4). The TLC of HCC obtained in the subgroup of BW > 60 kg was not discrepant with the quantitative evaluation of the aorta enhancement (Figure 5). And quantitative TLC at AP in Protocols A, B, C was lower than that in patients with lower BW (≤ 50 kg) in Protocol-D. In particular, TLC in Protocol-B was significantly lower than that in Protocol-D (difference: 17.00HU, *P* = 0.0249).

**Figure 6:**
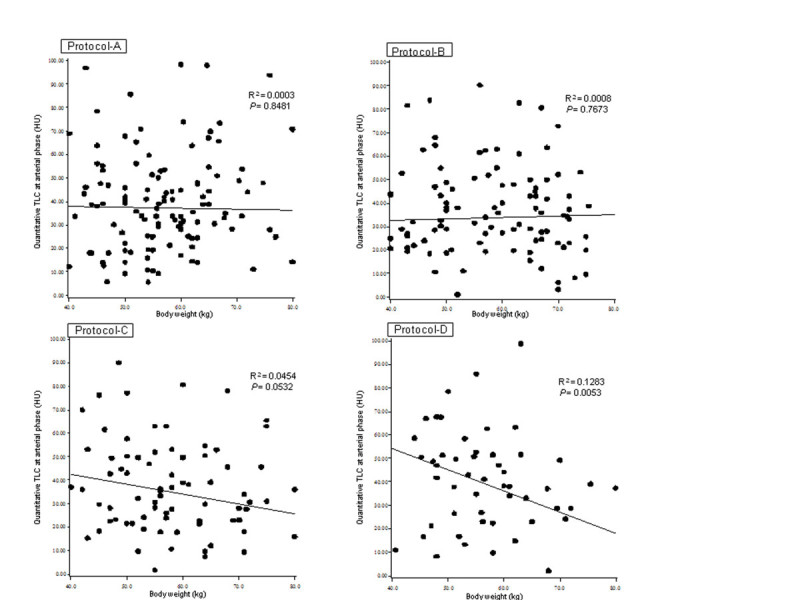
**Scatter plot of the relationship between body weight (BW; kg) and tumor liver contrast (TLC) at arterial phase (AP).** Protocol-**D** showed closer linear approximation between BW and TLC between 4 protocols, because R^2^ of Protocol-**D** was closer to 1 than those of Protocols **A**, **B**, **C**. Protocol-D showed significant inverse relationship between BW and TLC (*P* = 0.0053). HU; Hounsfield unit.

Scatter plot of the relationship between BW and TLC at AP was shown in Figure 6. R^2^ of Protocol-D (0.1283) was closer to 1 than those of Protocols A, B, C (Protocol-A; 0.0003, Protocol-B; 0.0008, and Protocol-C; 0.0454), and Protocol-D showed significant inverse relationship between BW and TLC (*P* = 0.0053).

### Safety

The CM was well tolerated by the patients. There were no clinically relevant changes in hemodynamic or laboratory parameters. No death or any adverse event leading to the discontinuation of a patient’s participation in the study was reported. Of the 417 patients who were investigated for adverse reactions and extravasation, a total of 6 (1.44%) reported adverse reactions, including sneezing in 1 patient (0.69%, 1/144) in Protocol-A, drug eruption in 1 patient and urticaria in 1 patient (1.70%, 2/118) in Protocol-B, eczema in 1 patient and nausea in 1 patient (2.30%, 2/87) in Protocol-C, and nausea in 1 patient (1.47%, 1/68) in Protocol-D. Both patients who had nausea recovered without treatment, and the rest of 4 patients (0.96%) were administered anti-allergy drugs such as adrenocortical steroid, antihistamine, glycyrrhizin for treatment of adverse reactions. A total of 3 (0.72%) patients reported extravasation; 1 patient (0.69%, 1/144) on Protocol-A, 1 (0.85%, 1/118) on Protocol-B, and 1 (1.15%, 1/87) on Protocol-C. But, there was no significant difference in the incidence and the trend of adverse reactions and extravasations among the 4 protocols. Moreover, we did not identify any concerns about safety in the constant injection duration method, although the infusion rate in patients with high BW would be increased by setting injection duration as constant.

## Discussion

Our study showed that in patients with higher BW (> 50 kg), protocols of BW-tailored dose of CM and/or fixed injection duration had higher tumor conspicuity to diagnose hypervascular HCCs, because they had higher tumor conspicuity and TLC of HCC than a fixed dose (100 mL) of CM. Whereas, the use of a CM with a concentration of 300 mgI/mL with fixed 100 mL was higher tumor conspicuity and TLC of HCC for dynamic hepatic CT in patients of BW ≤ 50 kg, since the volume of CM was injected in overload for those patients. To our knowledge, this study is the first prospective study evaluated the role of CM injection protocols for a large number of patients in community hospitals, although these results are theoretically presumable.

In 4 protocols of our study, we disclosed the relationship of BW and TLC at AP. R^2^ (0.1283) in Protocol-D showed closer linear approximation between BW and TLC, although the R^2^ was not high. This means that Protocol-D may not achieve diagnostically sufficient TLC in patients with higher BW. Protocol-D is not recommended for a liver CT, because injection protocol should be stable for all patients with wide range of BW.

Many patients are diagnosed in community hospitals. The optimal injection method on liver CT is required to detect HCC in not only academic organizations, but also community hospitals. From our results, we can call attention to staffs in the department of radiology worldwide including community hospitals in regard to the optimal injection protocol for HCC. We believe that liver dynamic CT protocols with BW-tailored dose of CM, fixed injection duration and scan timing at AP decided by bolus tracking should be recommended to obtain optimal HCC enhancement, because fixed injection duration can minimize the patients’ variables and can make the scan timing for each phase more uniform among all patients (Awai et al. 2004b; Bae 2003). However, the optimal BW-tailored dose of CM in patients with chronic liver diseases should be investigated to detect hypervascular HCC in the future. And scan delay at AP by bolus tracking should be more optimized in each hospital.

Awai et al. reported that 520 mgI/kg of contrast dose was not enough for liver enhancement in patients with chronic liver damage (Awai et al. 2004a), and Yamashita et al. reported that 600 mgI/kg of contrast dose is recommended for liver enhancement in patients with chronic liver damage (Yamashita et al. 2000). The mean contrast dose in our study was 543.6 to 593.9 mgI/kg (Table 3) in the 4 protocols. Thus, we believe that almost all cases had enough contrast dose in our study, except in patients with Protocol-D (mean contrast dose; 543.6 mgI/kg).

Injection rate is an important factor to detect hypervascularity of HCC at AP. Higher injection rate of CM allows higher HCC enhancement at AP, and TLC of HCC increases (Yanaga et al. 2007). In our study, mean of the injection flow rate in Protocol-A (3.79 mL/sec) was higher than other protocols (Table 3). In general, the higher injection flow rate works better to obtain higher detection rate of HCC, when other injection parameters are constant (Kim et al. 1995). A reason why Protocol-A did not achieve highest tumor conspicuity and TLC among the 4 protocols is that injection rate is only one factor for tumor conspicuity and TLC of HCC, and various other factors such as iodine dose, scan timing for AP relates to tumor conspicuity and TLC. In our study, injection duration, injected dose of CM per BW and scan timing at AP were different between 4 protocols. Thus the higher injection flow rate may be one reason that increases tumor conspicuity and TLC of HCC, although Protocol-A did not show higher tumor conspicuity and TLC than other protocols (Figures 3 and 5).

Kim et al. stated that an injection rate of 3 – 5 ml/sec took a mean 18 or 19 s to reach the threshold of 100 HU in the aorta (Kim et al. 1998). Sultana et al. reported an 18 s delay from bolus tracking to depict hypervascular HCC in the setting of trigger threshold level 100 HU in the aorta with an injection duration of 25 s by using 40 detectors-row CT (Sultana et al. 2007). Therefore, the optimal scan delay at AP from the initiation of CM injection is calculated as follows;

$$\begin{array}{l} \text{Scan}\;\text{delay}\;\mathrm{at}\;\mathrm{AP}=18\;s\;\left(\mathrm{or}\;19\;s\right)+18\;s\\ \;=36\;s\;\left(\mathrm{or}\;37\;s\right) \end{array}$$

This formula is applied in the case of a 25 s injection duration, thus the next formula is applied in the case of a 30 s injection duration

$$\begin{array}{l} \text{Scan}\;\text{delay}\;\mathrm{at}\;\mathrm{AP}=18\;s\;\left(\mathrm{or}\;19\;s\right)+18\;s+5\;s\\ \;=41\;s\;\left(\mathrm{or}\;42\;s\right) \end{array}$$

Moreover, when the injection duration is fixed, the peak enhancement times of aorta, portal vein, and liver constantly are approximately 10 s, 20 s, and 30 s after the any fixed injection durations, respectively (Ichikawa et al. 2006; Erturk et al. 2008). And the use of fixed injection duration (30 s) and scan delay of AP (approximately 40 s; 30 s + 10 s) after the start of CM injection are recommended to obtain sufficient peak enhancement value of hypervascular HCC (Ichikawa et al. 2006). As shown in the result of our study, median of the delay time in Protocols A and D at the AP was 40.0 s after the start of CM injection. This fact was based on the theory of “CM injection duration time + 10 s” for the AP on liver dynamic CT.

In Protocols B and C with bolus tracking system, the mean scan delay at AP was 34.92 s for Protocol-B and 32.77 s for Protocol-C, therefore they were early for the AP to depict hypervascular HCC. We thought that this is a valuable findings only through the Post-marketing surveillance by collecting data from the situation in the daily clinical settings in the community hospitals. This may be one of the causes of lower TLC in Protocols B and C than in Protocol-D in the subgroup of BW ≤ 50 kg (Figure 6). In addition, another cause is hypothesized that Protocol-D had superfluous iodine dose in regard to patients’ BW in the subgroup of BW ≤ 50 kg.

We believe that the scan delay for the AP in Protocols B and C used in daily clinical settings in the community hospitals was shorter than optimal. This may result in the lower tumor conspicuity and TLC in the qualitative and quantitative analysis. Therefore the trigger point of CM arrival and the scan delayed time after aortic arrival by using bolus tracking system should be optimized.

There are several potential limitations of our study design. First, our study includes heterogeneous protocols for dynamic liver CT, and characters of HCC are different between 4 protocols. Therefore, we collected a relatively large number of patients to overcome the variations among patients. Second, a bolus tracking system may not work as optimized scan delay in several hospitals, because the delay after bolus tracking for AP is shorter than other protocols without bolus tracking systems. From our results, we should warn diagnostic radiologists that liver dynamic CT has to be performed with an optimal time delay after the threshold of the bolus tracking system. Third, our fixed dose (100 mL) of 300 mgI/mL of iohexol appears to be inadequate for Western people. However, most of the patients in our study weighed less than 60 kg (mean, 58.1 kg), and 100 mL is still used in Japanese hospitals, so we chose this dose for Protocol-D. Our results should be more obvious in a Western population with a higher mean body weight of 70 kg or more. Despite these limitations, we believe it clinically significant that the finding on the protocols of BW-tailored dose of CM and/or fixed injection duration indicated by the data obtained from single institution was replicated also in daily clinical settings, especially to standardize CM injection protocol nationwide or worldwide.

In patients with higher BW (> 60 kg), protocols of BW-tailored dose of CM and/or fixed injection duration should be employed to diagnose hypervascular HCCs. Use of CM with constant 100 mL at any BW and fixed scan timing is not recommended for dynamic hepatic CT, except in patients with lower BW (≤ 50 kg). Moreover, when using bolus tracking system, its settings such as the trigger point and the scan delayed time should be optimized.

## Acknowledgments

The authors are grateful to all the patients, physicians, and co-medical staffs who participated in this study, and show great honor and sincere gratitude to the late Mr. Atsushi Hatcho, radiological technologist, for his contribution to planning, interpretation of the data, and the methodology of CT value measurement of this study.

## Authors’ original submitted files for images

Below are the links to the authors’ original submitted files for images. Authors’ original file for figure 1 Authors’ original file for figure 2 Authors’ original file for figure 3 Authors’ original file for figure 4 Authors’ original file for figure 5 Authors’ original file for figure 6

## References

[CR1] Awai K, Hori S (2003). Effect of contrast injection protocol with dose tailored to patient weight and fixed injection duration on aortic and hepatic enhancement at multidetector-row helical CT. Eur Radiol.

[CR2] Awai K, Inoue M, Yagyu Y, Watanabe M, Sano T, Nin S (2004). Moderate versus high concentration of contrast material for aortic and hepatic enhancement and tumor-to-liver contrast at multi-detector row CT. Radiology.

[CR3] Awai K, Hiraishi K, Hori S (2004). Effect of contrast material injection duration and rate on aortic peak time and peak enhancement at dynamic CT involving injection protocol with dose tailored to patient weight. Radiology.

[CR4] Bae KT (2003). Peak contrast enhancement in CT and MR angiography: when does it occur and why? Pharmacokinetic study in a porcine model. Radiology.

[CR5] Bae KT, Heiken JP, Brink JA (1998). Aortic and hepatic contrast medium enhancement at CT. Part I. Prediction with a computer model. Radiology.

[CR6] Bae KT, Heiken JP, Brink JA (1998). Aortic and hepatic contrast medium enhancement at CT. Part II. Effect of reduced cardiac output in a porcine model. Radiology.

[CR7] Berland LL, Lee JY (1988). Comparison of contrast media injection rates and volumes for hepatic dynamic incremented computed tomography. Invest Radiol.

[CR8] Brink JA, Heiken JP, Forman HP, Sagel SS, Molina PL, Brown PC (1995). Hepatic spiral CT: reduction of dose of intravenous contrast material. Radiology.

[CR9] Claussen CD, Banzer D, Pfretzschner C, Kalender WA, Schorner W (1984). Bolus geometry and dynamics after intravenous contrast medium injection. Radiology.

[CR10] Dean PB, Violante MR, Mahoney JA (1980). Hepatic CT contrast enhancement: effect of dose, duration of infusion, and time elapsed following infusion. Invest Radiol.

[CR11] Erturk SM, Ichikawa T, Sou H, Tsukamoto T, Motosugi U, Araki T (2008). Effect of duration of contrast material injection on peak enhancement times and values of the aorta, main portal vein, and liver at dynamic MDCT with the dose of contrast medium tailored to patient weight. Clin Radiol.

[CR12] Han JK, Kim AY, Lee KY, Seo JB, Kim TK, Choi BI (2000). Factors influencing vascular and hepatic enhancement at CT: experimental study on injection protocol using a canine model. J Comput Assist Tomogr.

[CR13] Heiken JP, Brink JA, McClennan BL, Sagel SS, Crowe TM, Gaines MV (1995). Dynamic incremental CT: effect of volume and concentration of contrast material and patient weight on hepatic enhancement. Radiology.

[CR14] Ichikawa T, Erturk SM, Araki T (2006). Multiphasic contrast-enhanced multidetector-row CT of liver: contrast-enhancement theory and practical scan protocol with a combination of fixed injection duration and patients' body-weight-tailored dose of contrast material. Eur J Radiol.

[CR15] Kim T, Murakami T, Oi H, Matsushita M, Kishimoto H, Igarashi H (1995). Detection of hypervascular hepatocellular carcinoma by dynamic MRI and dynamic spiral CT. J Comput Assist Tomogr.

[CR16] Kim T, Murakami T, Takahashi S, Tsuda K, Tomoda K, Narumi Y (1998). Effects of injection rates of contrast material on arterial phase hepatic CT. AJR Am J Roentgenol.

[CR17] Kondo H, Kanematsu M, Goshima S, Tomita Y, Miyoshi T, Hatcho A (2008). Abdominal multidetector CT in patients with varying body fat percentages: estimation of optimal contrast material dose. Radiology.

[CR18] Kormano M, Partanen K, Soimakallio S, Kivimaki T (1983). Dynamic contrast enhancement of the upper abdomen: effect of contrast medium and body weight. Invest Radiol.

[CR19] Sultana S, Awai K, Nakayama Y, Nakaura T, Liu D, Hatemura M (2007). Hypervascular hepatocellular carcinomas: bolus tracking with a 40-detector CT scanner to time arterial phase imaging. Radiology.

[CR20] Tomemori T, Yamakado K, Nakatsuka A, Sakuma H, Matsumura K, Takeda K (2001). Fast 3D dynamic MR imaging of the liver with MR SmartPrep: comparison with helical CT in detecting hypervascular hepatocellular carcinoma. Clin Imaging.

[CR21] Walkey MM (1991). Dynamic hepatic CT: how many years will it take ’til we learn?. Radiology.

[CR22] Yagyu Y, Awai K, Inoue M, Watai R, Sano T, Hasegawa H (2005). MDCT of hypervascular hepatocellular carcinomas: a prospective study using contrast materials with different iodine concentrations. AJR Am J Roentgenol.

[CR23] Yamashita Y, Komohara Y, Takahashi M, Uchida M, Hayabuchi N, Shimizu T (2000). Abdominal helical CT: evaluation of optimal doses of intravenous contrast material–a prospective randomized study. Radiology.

[CR24] Yanaga Y, Awai K, Nakayama Y, Nakaura T, Tamura Y, Funama Y (2007). Optimal dose and injection duration (injection rate) of contrast material for depiction of hypervascular hepatocellular carcinomas by multidetector CT. Radiat Med.

