# Supplementary figures and images for: The efficacy of contrast protocol in hepatic dynamic computed tomography: multicenter prospective study in community hospitals
Source: Springerplus. 2013 Jul 31;2(1):367. doi: 10.1186/2193-1801-2-367 (PMC3742842; doi:10.1186/2193-1801-2-367)

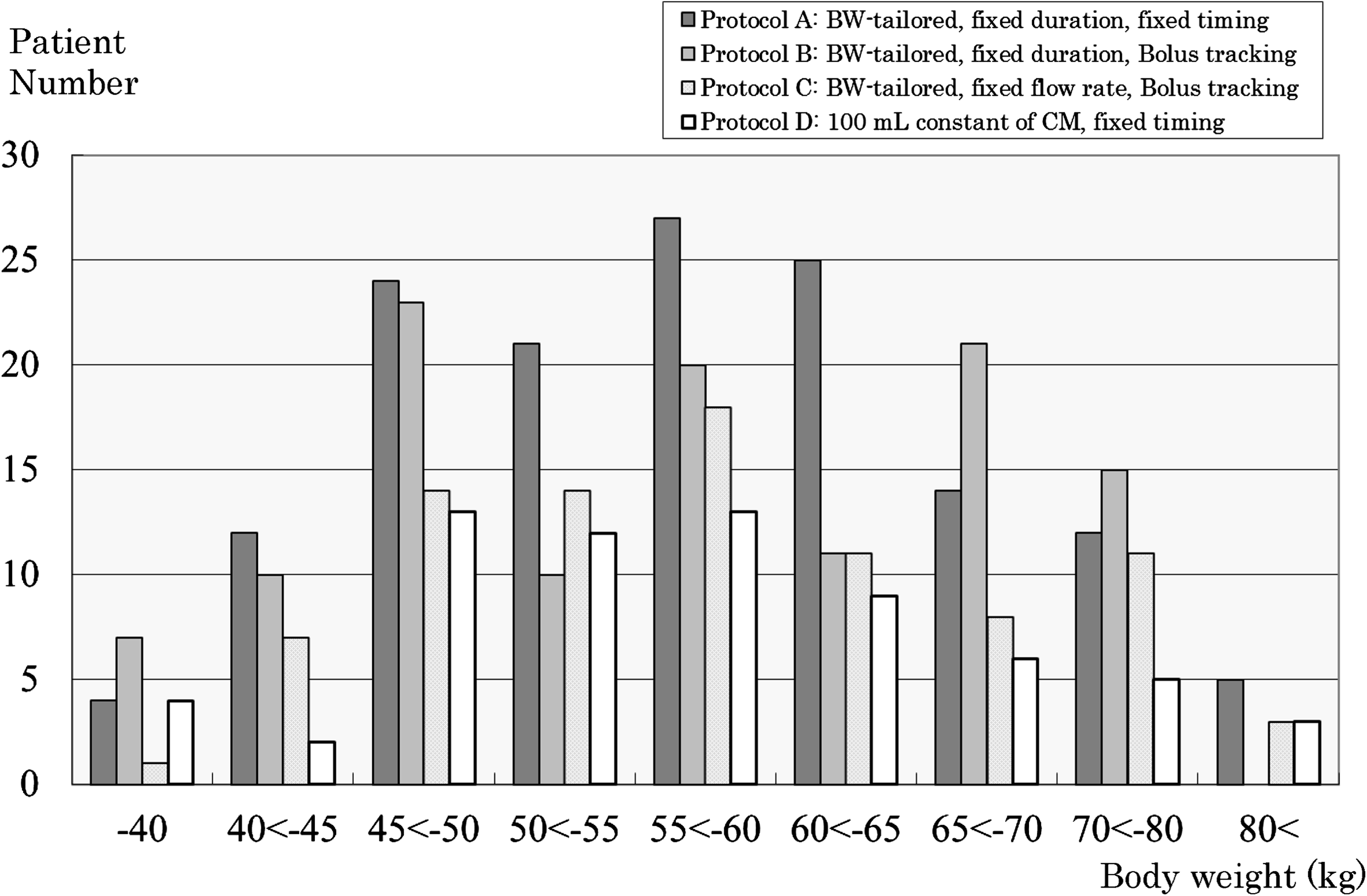

Supplement: Supplementary file 1 — Authors’ original file for figure 1 [file 40064_2013_440_MOESM1_ESM.tiff]

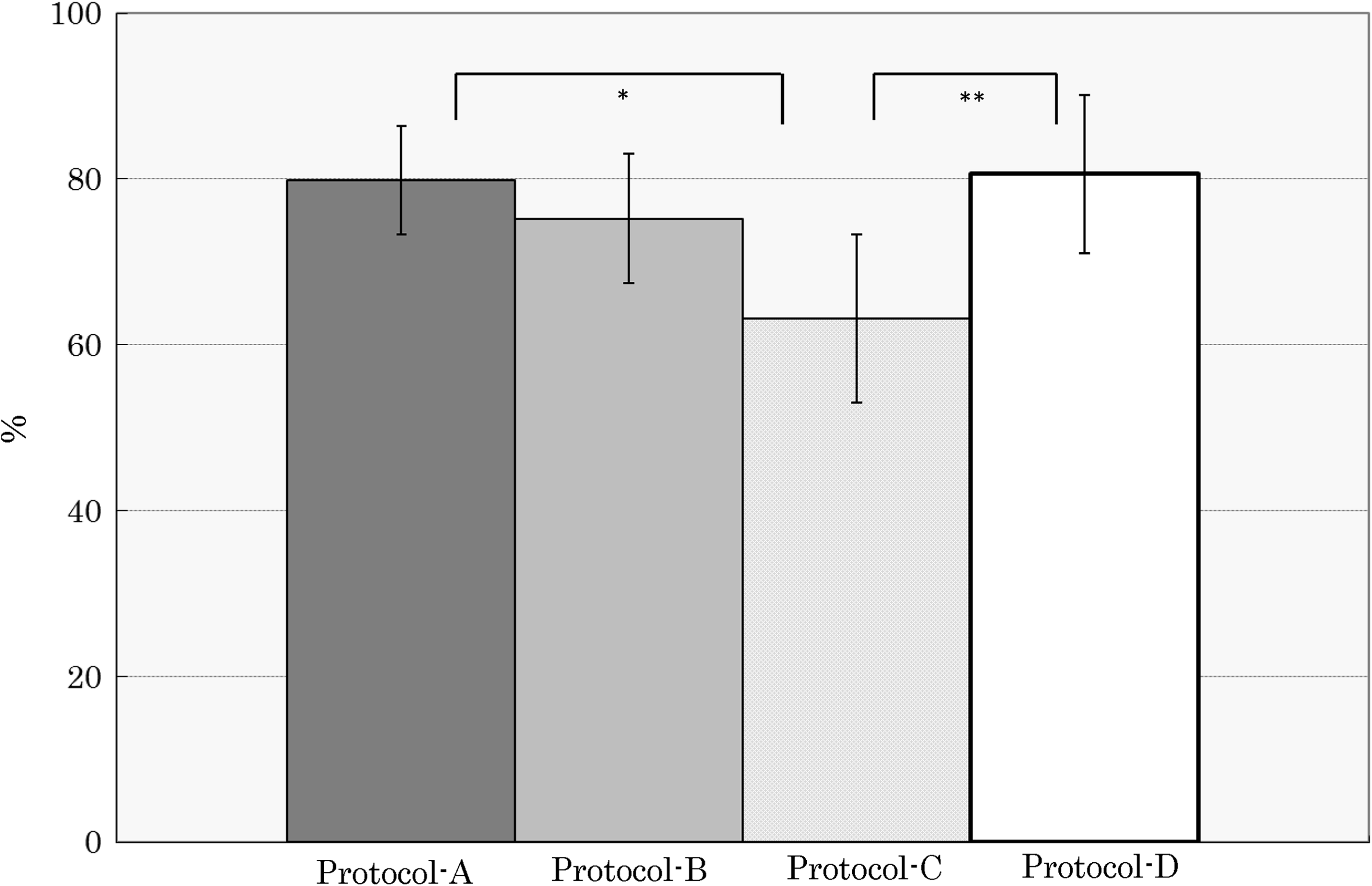

Supplement: Supplementary file 2 — Authors’ original file for figure 2 [file 40064_2013_440_MOESM2_ESM.tiff]

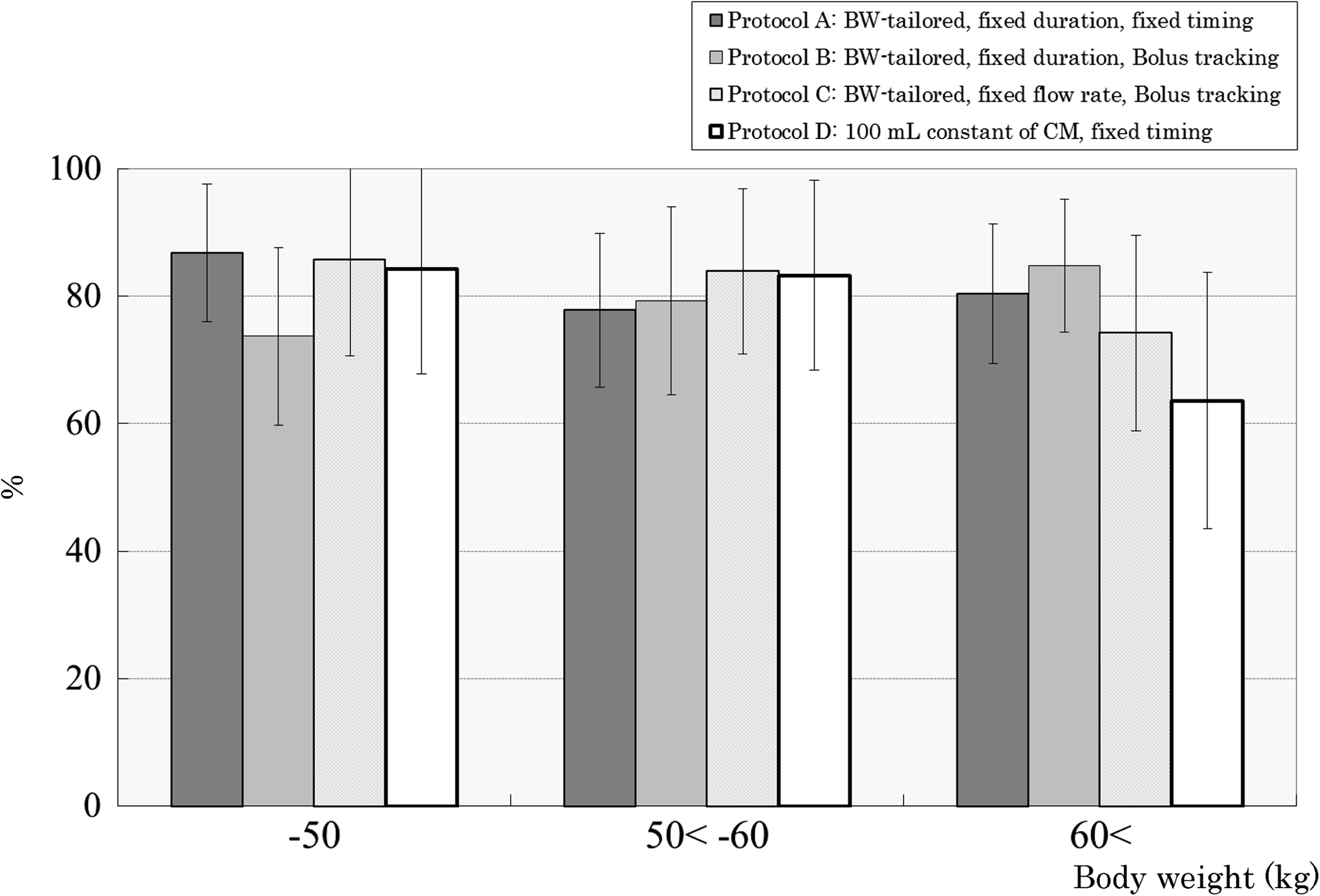

Supplement: Supplementary file 3 — Authors’ original file for figure 3 [file 40064_2013_440_MOESM3_ESM.tiff]

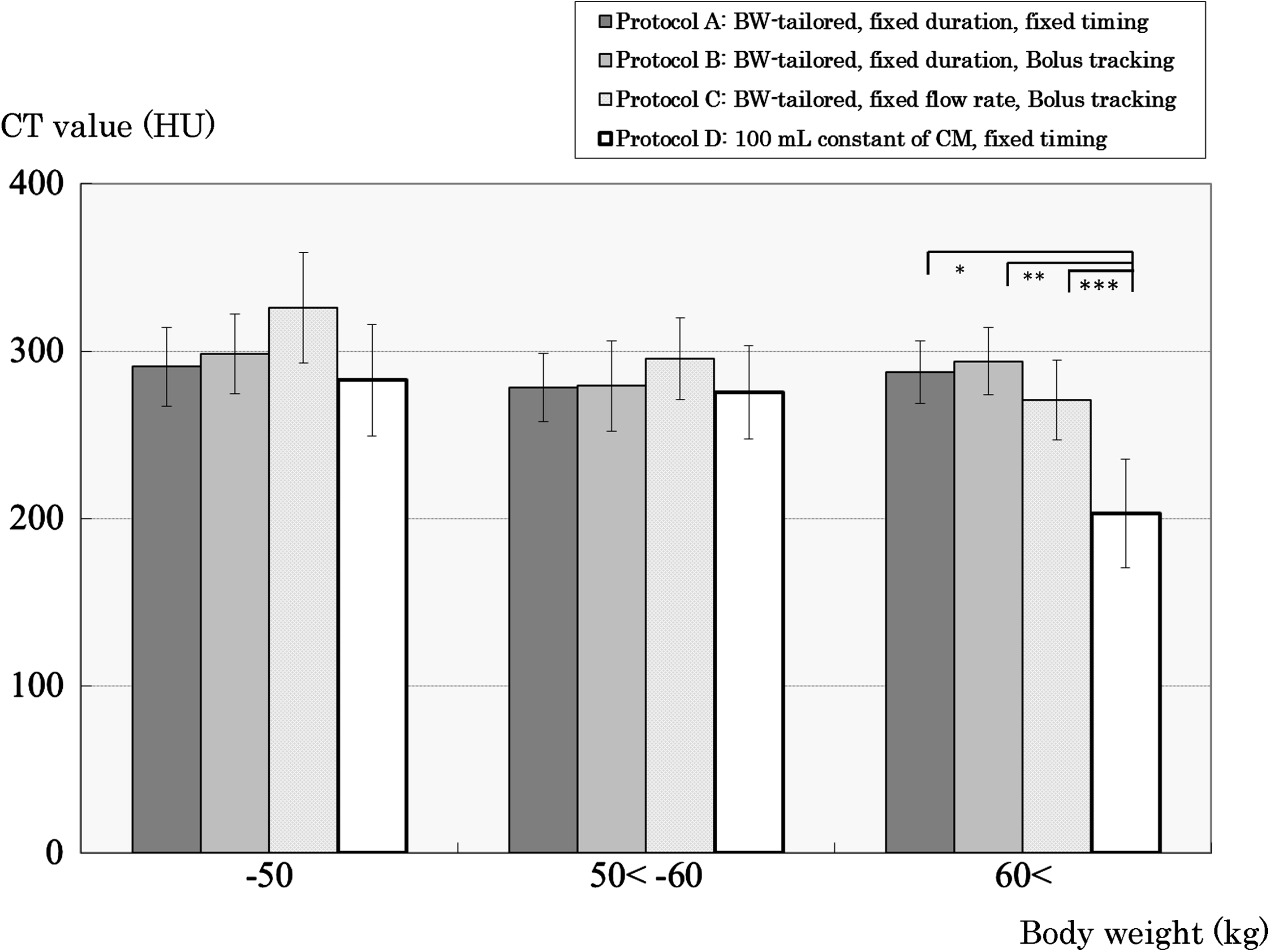

Supplement: Supplementary file 4 — Authors’ original file for figure 4 [file 40064_2013_440_MOESM4_ESM.tiff]

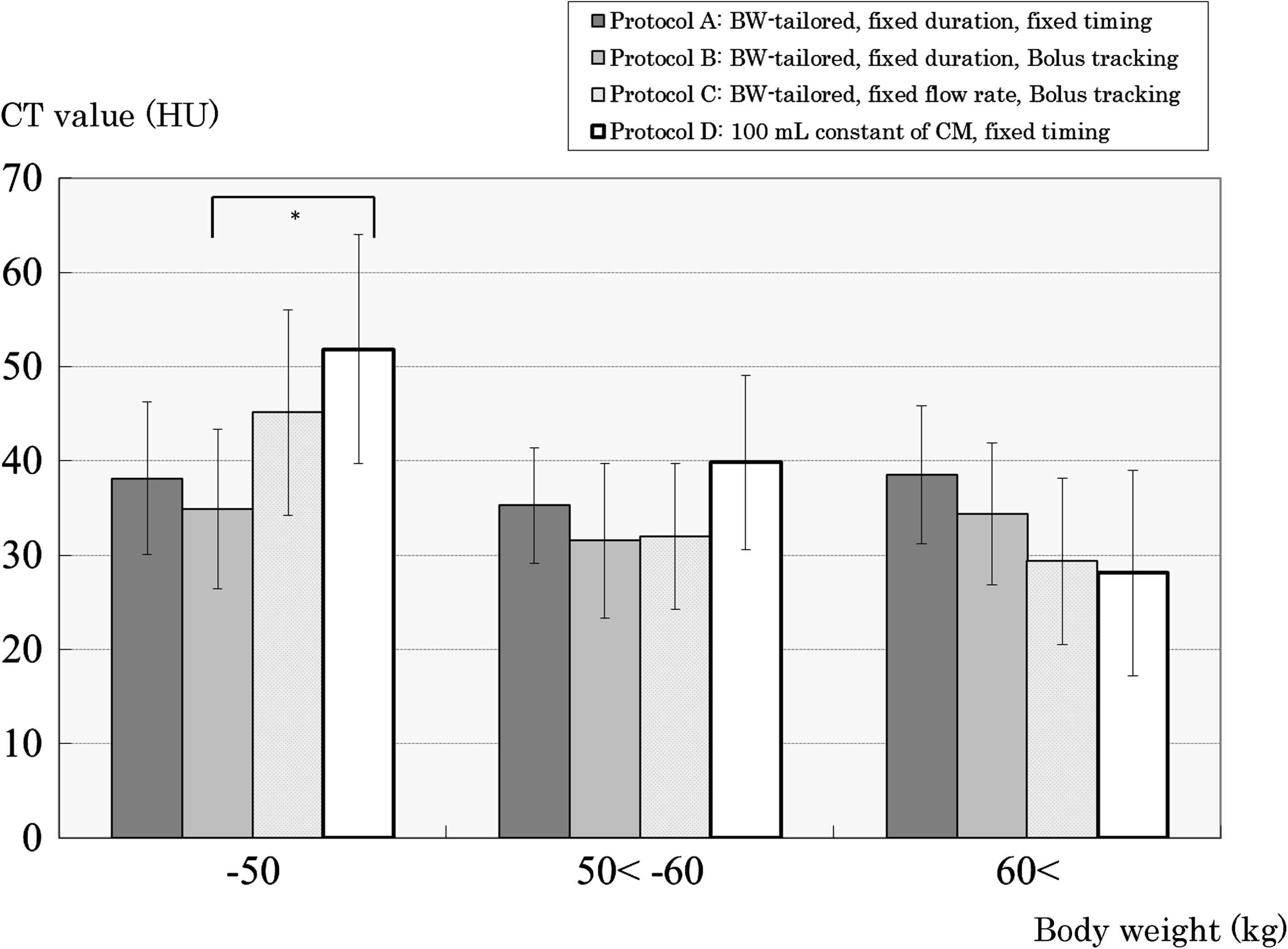

Supplement: Supplementary file 5 — Authors’ original file for figure 5 [file 40064_2013_440_MOESM5_ESM.tiff]

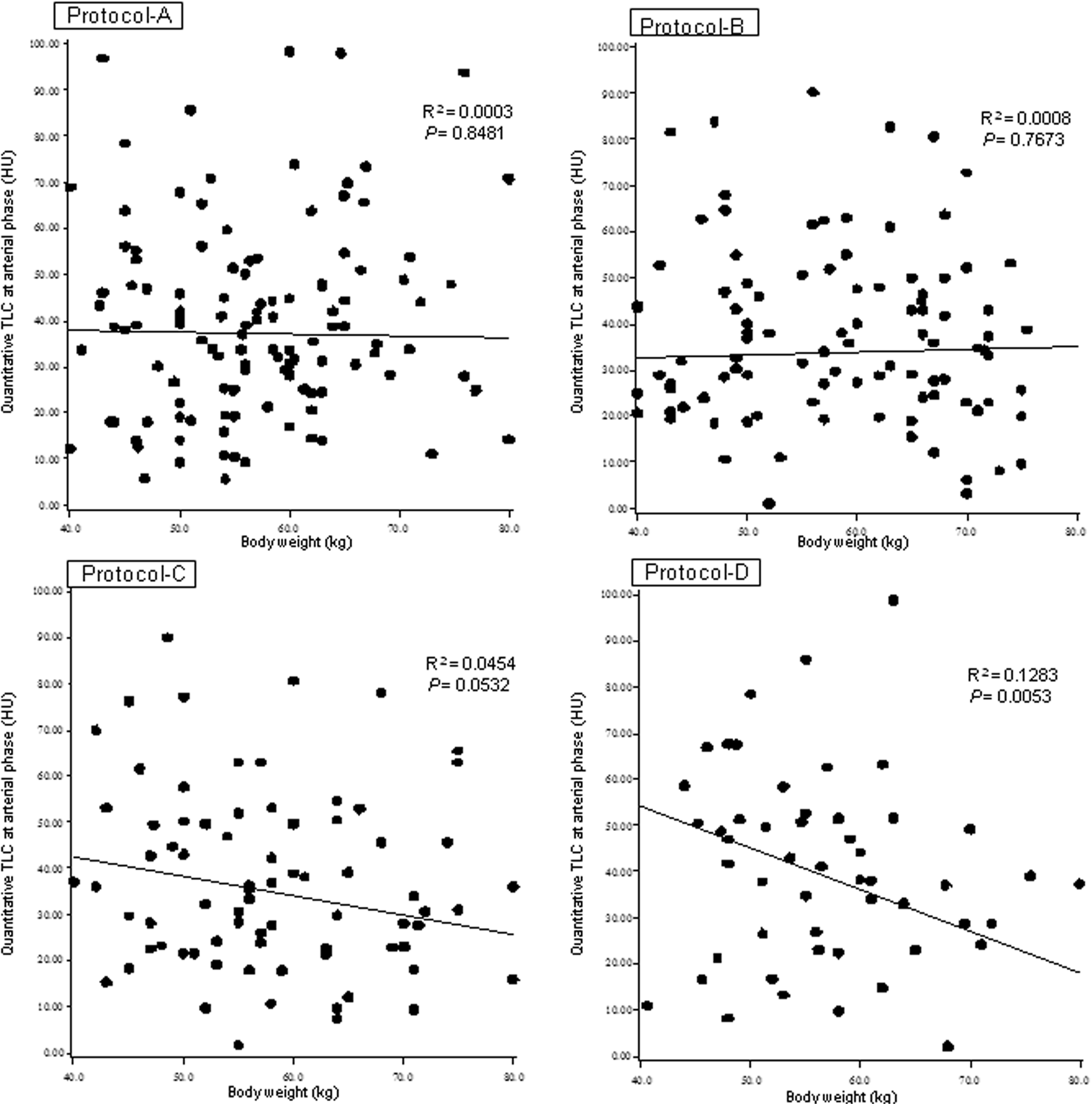

Supplement: Supplementary file 6 — Authors’ original file for figure 6 [file 40064_2013_440_MOESM6_ESM.tif]
